# Unlocking Hidden Teeth: A Pediatric Case of Surgical Exposure of Maxillary Central Incisors

**DOI:** 10.7759/cureus.62140

**Published:** 2024-06-11

**Authors:** Rashi Rathi, Himani Parakh, Nilima R Thosar, Meenal S Pande, Aakriti Chandra, Ramakrishna Yeluri, Neha Pankey, Vedant M Dhatrak

**Affiliations:** 1 Dentistry, Sharad Pawar Dental College and Hospital, Datta Meghe Institute of Higher Education and Research, Wardha, IND; 2 Pediatric and Preventive Dentistry, Sharad Pawar Dental College and Hospital, Datta Meghe Institute of Higher Education and Research, Wardha, IND; 3 Pediatric Dentistry, Sharad Pawar Dental College and Hospital, Datta Meghe Institute of Higher Education and Research, Wardha, IND

**Keywords:** pediatric dentistry, general anesthesia, surgical exposure, central incisor, unerupted teeth

## Abstract

Failure in the eruption of the maxillary incisor necessitates a precise diagnosis and treatment regimen. Impaction can have a number of causes such as physical obstacles in the eruption path, discrepancy in the length of the dental arch, and tooth anomaly. Delayed eruption of anterior teeth can result in a number of issues such as malocclusion and psychological discomfort. In many cases, if the intervention is not done at an early stage, complex orthodontic intervention is required after surgical exposure to enable appropriate eruption. This case report is of a nine-year-old child with unerupted maxillary central incisors. The child was treated surgically for incisor exposure under general anesthesia (GA). Both functional and aesthetic considerations made this surgical procedure necessary. GA was administered due to the negative behavior of the child in a dental setting. Hence, it ensured patient comfort and cooperation. Follow-up examinations showed satisfactory progress in the eruption of the teeth with no complications.

## Introduction

The impaction of deciduous teeth is a rare event. The appropriate health care of a patient may be significantly impacted by delayed tooth eruption. Unerupted or clinical absence of maxillary central incisors can have a significant impact on the dental as well as facial aesthetics of an individual; thus, prompt multidisciplinary management is indicated as unerupted incisors can lead to functional- and appearance-related distress. The missing upper incisors are considered unsightly, which could negatively impact an individual's social well-being and self-esteem. Also, it hampers phonetics since it has an effect on these teeth on the articulation of consonant speech sounds [1, 2]. In children aged 5 to 12 years, the prevalence of unerupted permanent maxillary incisors is 0.13% [3], with Asian races having a higher incidence [4]. The primary etiological factors that cause delayed eruption of maxillary permanent incisors can be classified into two main groups: hereditary and environmental factors. Hereditary causes are cleft lip and palate, cleidocranial dysostosis, odontoma, abnormal tooth/tissue ratio, and generalized delayed eruption. Similarly, the various environmental factors that can affect the eruption of incisors include trauma, cystic formation, endocrine problems, early loss or extraction of primary teeth (with or without space loss), retained deciduous teeth, and bone disease. The various treatment modalities currently available, according to the literature, for unerupted /impacted maxillary central incisors are as follows: continuous follow-up and evaluation, removal of retained primary tooth, creating and maintaining sufficient mesial and distal space, surgical orthodontic recovery, surgical exposure, and laser treatment. The following report presents a case of an unerupted maxillary central incisor in a nine-year-old child in whom the treatment was carried out under general anesthesia (GA) because of the highly uncooperative nature of the child.

## Case presentation

**Figure 1:**
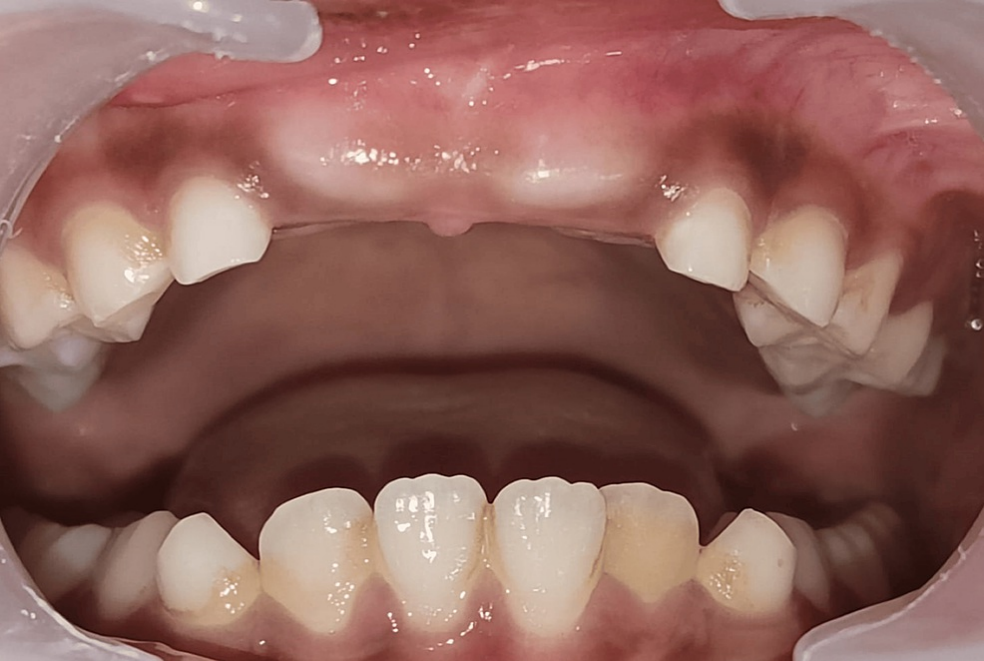
Unerupted right and left permanent maxillary central incisors (11, 21)

**Figure 2:**
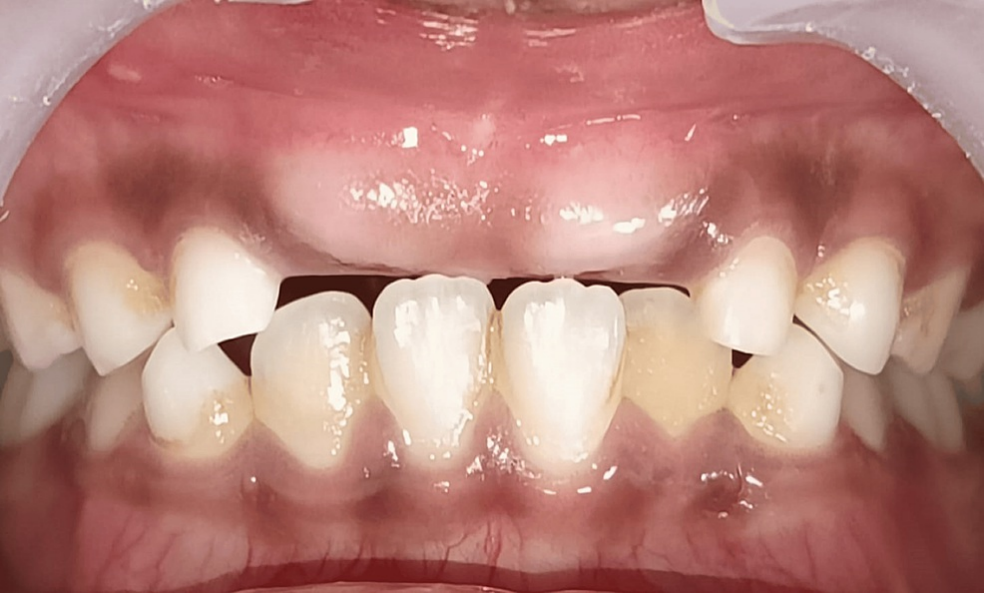
Pre-eruptive bulge seen as a clinical sign of unerupted central incisors (11, 21)

**Figure 3:**
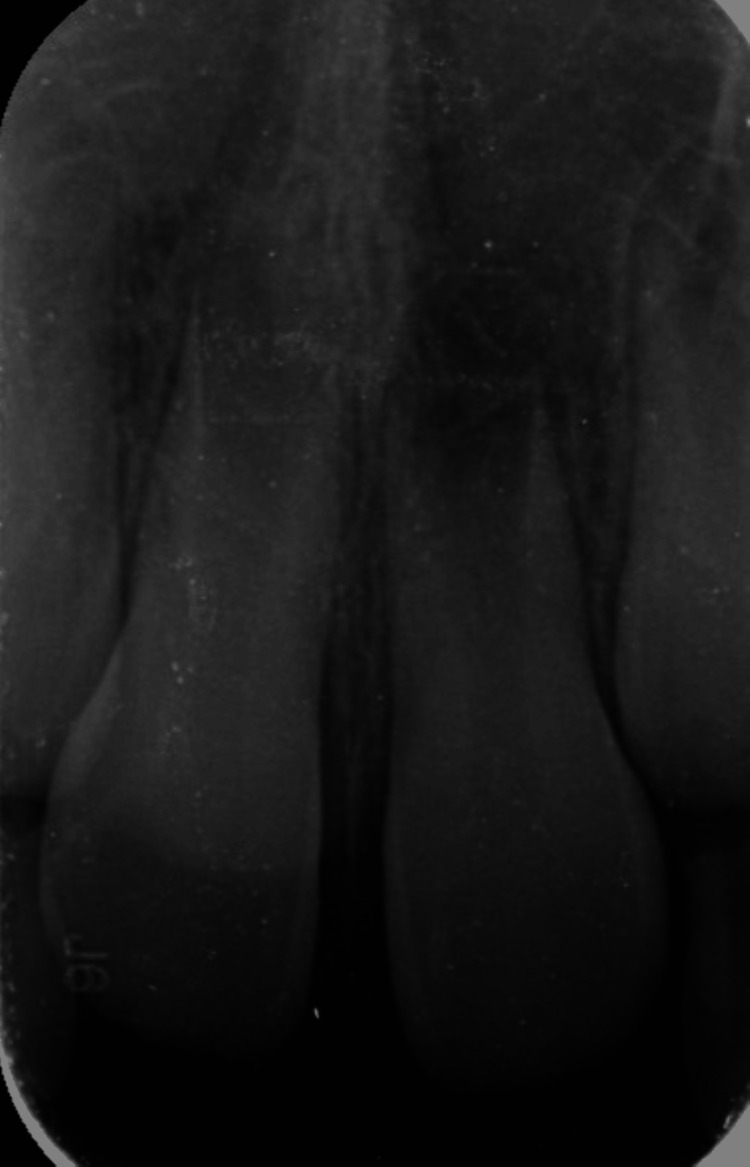
Digital radiograph (radiovisiography) confirming the presence of the embedded central incisors (11, 21)

A nine-year-old child was reported to the Department of Pediatric and Preventive Dentistry with a chief complaint of the absence of teeth in the upper front region of the jaw for one to two years. The boy was concerned with aesthetic displeasure and social awkwardness, which restricted his everyday activities. While recording, medical, family, and dental history presented with no significant findings. The patient, however, presented with an uncooperative behavior according to Frankel’s negative behavior. On inspection, intraorally, all the soft and hard tissue examinations were normal except for the presence of the missing maxillary central incisors. The pre-eruptive bulge was seen on the buccal aspect of the gingiva with respect to 11 and 21, which was pale pink (Figures 1-2). On palpation, the bulge was hard in consistency, indicating the presence of impacted/unerupted maxillary central incisors. On radiographic investigation, no hard tissue was seen surrounding the crowns of permanent maxillary incisors, though clinically, it was seen that a thick layer of soft tissue surrounded the crown. On radiograph, Nolla’s stage 7 was seen, i.e., 2/3 of root development has been done with both the central incisors (Figure 3).

Treatment

The planned treatment for this case was surgical exposure of the teeth. However, as the child was classified as definitively negative according to Frankl's behavior rating scale, this case was planned under GA. The preoperative health evaluation of the child was done, which included a physical status evaluation and measurements of age, height, and weight. A medical history was also taken, including physical anomalies and neurological impairments that may raise the risk for airway blockage, adverse drug reactions, medication/drug histories, previous relevant hospitalizations, and family history. A review of systems was conducted, paying particular attention to abnormalities in cardiac, hepatic, pulmonary, and renal function. This review included assessments of blood group, hemoglobin (Hb), total leukocyte count (TLC), platelet count, mean corpuscular volume (MCV), mean corpuscular hemoglobin (MCH), mean corpuscular hemoglobin concentration (MCHC), serum bilirubin, total protein (including albumin and globulin), serum urea, creatinine, international normalized ratio (INR), prothrombin time (PT), and activated partial thromboplastin time (APTT). Determination of vital signs such as temperature, blood pressure, heart rate, and respiratory rate was performed. A physical examination, including a focused evaluation of the airway to ascertain the elevated risk of airway obstruction, was conducted. This included an analysis of the airway in cooperative children based on the Mallampati classification [5].

Following a standard protocol, GA was induced with a face mask containing 2% sevoflurane after injecting propofol. The anesthesia was maintained through intravenous propofol administration. The patient was intubated, and a pediatric mouth molt was used to obtain surgical access to the patient's mouth. Salivation had been managed with a saliva ejector, and the pharyngopalatine region was covered with moist, sterile gauze to prevent aspiration. An injection of 2% lignocaine mixed with epinephrine was used as a local anesthetic.

**Figure 4:**
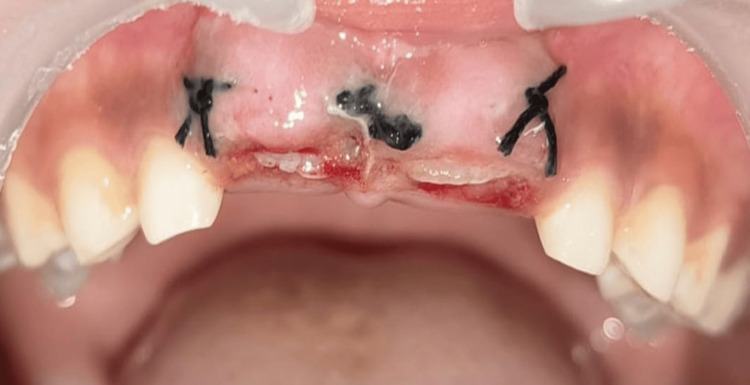
Surgical exposure of central incisors followed by suture placement

Clinical and radiological evaluations served as the basis for the treatment approach. Two vertical incisions were given on each tooth, i.e., right (11) and left (21), on the mesial and distal side of the bulge, and one horizontal incision on the buccal aspect using #15 blade and number 4 Bard-Parker (BP) handle. The mucoperiosteal flap was reflected 2 mm cervically till the incisal edges of the maxillary central incisors became visible. Then, simple interrupted sutures using 4-0 silk were given (Figure 4).

The two central incisors were then exposed and allowed to erupt now physiologically. After the dental treatment, atropine and neostigmine were used to reverse the muscle relaxation action induced during GA.

**Figure 5:**
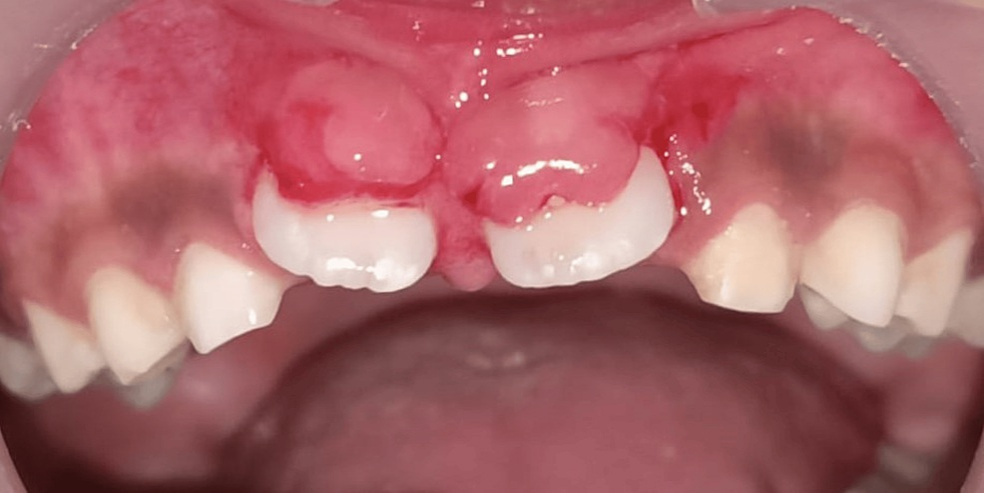
Eruptive movement of central incisors seen within one week of surgical exposure

The patient was then monitored in the Pediatric Intensive Care Unit. Also, the patient was told to consume a soft diet and maintain oral hygiene. After a week, the patient was recalled for follow-up, and it was observed that maxillary central incisors had their eruptive movement progressed occlusally by 2 mm (Figure 5).

## Discussion

It is evident that children and teenagers frequently exhibit delayed eruption of their maxillary central incisors in the dental clinic. A failed eruption will impact the growing occlusion and can impact the child's psychological growth. This condition may cause malocclusion, poor dental and facial aesthetics, localized space loss, and difficulty speaking. If at all possible, impacted maxillary incisor teeth should ideally be positioned inside the dental arch as soon as possible to provide the patient with the best possible dental, functional, cosmetic, and psychological outcomes. Permanent maxillary incisors that are unerupted or impacted might result from a combination of environmental and developmental factors. Patients who exhibit an eruption disturbance of the permanent upper incisors and are between the ages of seven and nine ought to be referred for suitable treatment.

A significant body of literature exists regarding the treatment of unerupted upper incisors. According to Becker, the suggested steps in the treatment sequence are opening the space to encourage a proper eruption process, surgical obstacle removal to reveal the unerupted tooth, and orthodontic traction followed by alignment [6].

There is debate regarding the ideal time for the removal of the obstruction. Mason et al. observed that spontaneous eruption occurred in 72% of immature incisors after early removal of associated obstruction, whereas 63% of mature incisors needed further surgery. Their findings suggested the likelihood of spontaneous eruption is increased when a blockage is removed early before the adjacent incisor tooth erupts [7]. However, in order to prevent harm to the tooth's apical region, Alacam and Bani suggested delaying treatment until the adjacent incisor had reached maturity [8].

The removal of any obstruction that might prevent eruption and the provision of adequate space within the dental arch are the fundamentals for managing unerupted maxillary incisors, according to recent guidelines. Other factors that may contribute to this condition include the patient's age, medical history, treatment compliance, the cause, and the site of the delayed erupted incisor [9]. In retrospective research, Leyland et al. discovered that, given space available, between 49 and 91% of maxillary permanent incisors spontaneously erupted within 18 months after the removal of supernumerary teeth [10].

The major cause of obstruction is early loss of primary incisors causing the underlying soft tissue to turn into thick fibrous band due to which obstruction is created in eruption of permanent maxillary central incisors.

In this case, the child’s chronological age was nine years, while his dental age was eight years. The permanent maxillary central incisors were in Nolla’s stage 7, i.e., 2/3 of root development has been completed. Clinically, a pre-eruptive bulge was seen and palpable of the permanent maxillary central incisors, which was pale pink in color.

The treatment planned was surgical excision; however, the patient's uncooperative behavior made it difficult for the dentist to carry out the procedure chair-side under local anesthesia. Thus, GA induction was then planned after proper pre-operative evaluation. GA is the recommended course of treatment for medically sound patients who have severe dental anxiety or are extremely uncooperative. It is also advised for patients with complicated medical issues, as well as for very young children who require extensive dental procedures due to advanced full-mouth caries and need comprehensive dental care [11]. The induction of GA, in this case, has provided a number of benefits. These included the elimination of the need for patient cooperation, the patient's unconscious state and lack of pain response, a degree of amnesia, and the ability to titrate medication to the ideal dosage. Additionally, it allowed for the tracking of spontaneous eruption during follow-up visits. A two-month follow-up was taken, and it was noticed that the permanent maxillary central incisors erupted without the need for orthodontic treatment [12].

## Conclusions

In conclusion, the permanent maxillary central incisors spontaneously erupted within six months of follow-up after the reflection of the mucoperiosteal flap covering the permanent central incisors due to early exfoliation of the deciduous central incisors managed surgically under GA without any orthodontic interception.

The multidisciplinary approach to treating an impacted central incisor allowed the functional and aesthetic recovery of the patient in a short amount of time, which reduces the psychological impact caused by the lack of an anterior tooth.
